# A Role for P-selectin and Complement in the Pathological Sequelae of Germinal Matrix Hemorrhage

**DOI:** 10.21203/rs.3.rs-2617965/v1

**Published:** 2023-02-28

**Authors:** Devin Hatchell, Mohammed Alshareef, Tyler Vasas, Silvia Guglietta, Davis Borucki, Chunfang Guo, Khalil Mallah, Ramin Eskandari, Stephen Tomlinson

**Affiliations:** Medical University of South Carolina; Medical University of South Carolina; Medical University of South Carolina; Medical University of South Carolina; Medical University of South Carolina; Medical University of South Carolina; Medical University of South Carolina; Medical University of South Carolina; Medical University of South Carolina

**Keywords:** Germinal Matrix Hemorrhage, Complement, P-selectin, Neuroinflammation, Microglia

## Abstract

**Background:**

Germinal Matrix Hemorrhage is a devastating disease of pre-term infancy commonly resulting in post-hemorrhagic hydrocephalus, periventricular leukomalacia, and subsequent neurocognitive deficits. We demonstrate vascular expression of the adhesion molecule P-selectin after GMH and investigate a strategy to specifically target complement inhibition to sites of P-selectin expression to mitigate the pathological sequelae of GMH.

**Methods:**

We prepared two fusion proteins consisting of different anti-P-selectin single chain antibodies (scFv’s) linked to the complement inhibitor Crry. One scFv targeting vehicle (2.12scFv) blocked the binding of P-selectin to its PSGL-1 ligand expressed on leukocytes, whereas the other targeting vehicle (2.3scFv) bound P-selectin without blocking ligand binding. Post-natal mice on day 4 (P4) were subjected to collagenase induced-intraventricular hemorrhage and treated with 2.3Psel-Crry, 2.12Psel-Crry, or vehicle.

**Results:**

Compared to vehicle treatment, 2.3Psel-Crry treatment after induction of GMH resulted in reduced lesion size and mortality, reduced hydrocephalus development, and improved neurological deficit measurements in adolescence. In contrast, 2.12Psel-Crry treatment resulted in worse outcomes compared to vehicle. Improved outcomes with 2.3Psel-Crry were accompanied by decreased P-selectin expression, and decreased complement activation and microgliosis. Microglia from 2.3Psel-Crry treated mice displayed a ramified morphology, similar to naïve mice, whereas microglia in vehicle treated animals displayed a more ameboid morphology that is associated with a more activated status. Consistent with these morphological characteristics, there was increased microglial internalization of complement deposits in vehicle compared to 2.3Psel-Crry treated animals, reminiscent of aberrant C3-dependent microglial phagocytosis that occurs in other (adult) types of brain injury. Also, following systemic injection, 2.3Psel-Crry specifically targeted to the post-GMH brain. Likely accounting for the unexpected finding that 2.12Psel-Crry worsens outcome following GMH was the finding that this construct interfered with coagulation in this hemorrhagic condition, and specifically with heterotypic platelet-leukocyte aggregation, which express P-selectin and PSGL-1, respectively.

**Conclusion:**

GMH induces expression of P-selectin, the targeting of which with a complement inhibitor protects against pathogenic sequelae of GMH. A dual functioning construct with both P-selectin and complement blocking activity interferes with coagulation and worsens outcomes following GMH, but has potential for treatment of conditions that incorporate pathological thrombotic events, such as ischemic stroke.

## Background

Germinal Matrix Hemorrhage (GMH) is a devastating disease of neonates caused by disruption of fragile vasculature within the subventricular zone (SVZ), the site of origin for cerebral precursor cells of both gray and white matter [1, 2]. GMH is a relatively common neurologic pathology in neonates, estimated at 3.5 per 1000 live births [3], and its occurrence is strongly associated with pre-term birth (< 32 weeks) and low birth weight (< 1500 g) [2, 4]. GMH involves a primary injury that cannot be treated by intervention due to the immediate mechanical trauma of a hemorrhagic mass, followed by a secondary progressive injury that extends into neighboring tissue [5]. A major pathologic sequela secondary to GMH is the onset and progression of post-hemorrhagic hydrocephalus (PHH), which is associated with a robust inflammatory response [6–8]. The complement system plays a central cascading role in orchestrating inflammatory responses. In the general setting of brain injury, complement activation products (C3 opsonins, the anaphylatoxins C3a and C5a, and C5b-9) have been strongly implicated in the progression of secondary injury [9–12], although the role of complement in the progression of secondary injury post-GMH is not well characterized [5, 13, 14].

Another important component of an inflammatory response is the expression of adhesion molecules on activated vasculature. Endothelial expression of the adhesion molecule P-selectin plays an important role in the recruitment of leukocytes, in particular neutrophils, to sites of inflammation [15, 16]. Furthermore, there is a dynamic relation between complement activation and P-selectin expression, with complement activation products able to upregulate P-selectin expression, and P-selectin able to directly activate complement [17–21]. Here we characterize two fusion proteins consisting of a P-selectin targeting moiety (an anti-P-selectin single chain antibody fragment, scFv) linked to a complement inhibitor (Crry) in our recently characterized GMH model [5]. One of these constructs, 2.12Psel-Crry, not only delivers Crry to sites of inflammation and P-selectin expression, but also blocks the interaction of P-selectin with its leukocyte counter-receptor, PSGL-1. The other construct, 2.3Psel-Crry, similarly targets Crry to sites of P-selectin expression but does not interfere with the binding of P-selectin to PSGL-1. The complement inhibitor Crry inhibits all complement pathways at the proximal C3 activation step.

## Materials And Methods

### Study Design

This study used five animal treatment groups: Wild-type naïve (no GMH injury and no treatment), Sham (endotoxin free PBS injection in the SVZ in place of collagenase and no treatment), Vehicle (collagenase injection in the SVZ and intraperitoneal (ip) PBS treatment), and Psel-Crry treated (collagenase injection in the SVZ and ip treatment of either 2.12Psel-Crry or 2.3Psel-Crry). Prior to surgical procedures for GMH induction, animal breeders were randomly assigned to groups. Randomization was performed by an external lab member and was dependent on liter sizes at post-natal day 1 (P1) of life in order to accommodate adequate numbers across groups. All GMH injuries were performed by guided injection of collagenase into the subventricular zone (SVZ) on post-natal day 4 (P4) as previously described [5]. For testing, scorer was blinded to group allocations. Following injection, pups were placed on a heating pad for 30 minutes and then reunited with the mother. The total handling time of pups away from the mother was approximately 45 minutes. Animals were then monitored for an additional 60 minutes to ensure care of the pups by the mother. Animals were excluded if they died within 24 hours of surgery (< 10% of animals). Study endpoints were P14 (10 days post-injury) for acute outcome analyses, including histology and behavioral testing, P30 for MRI analysis, and P45 for analysis of animal survival and for behavioral tasks.

## Animal Husbandry and Care

The Institutional Animal Care and Use Committee (IACUC) at the Medical University of South Carolina approved all animal procedures. Wild-type male and female C57Bl/J mice (Jackson Laboratory, ME, USA) were obtained at age P30 and acclimatized for 1 week. Animals were then mated in pairs. Cages were cleaned weekly and new bedding provided. All mice housed in the facility were exposed to 12 hours light/dark cycles and received access to food and water ad libitum, while pregnant females received a high fat diet as recommended by the institutional veterinarian. All tests and experiments were conducted during the light cycle. Pregnancy and litter checks were performed daily. Males were separated to another cage on day of pup injury due to propensity of male mice to kill injured offspring. All animals were returned to the mouse housing facility following procedures and tests.

## Construction, Expression, and In Vitro Characterization of Recombinant Proteins

2.3Psel-Crry and 2.12Psel-Crry are recombinant fusion proteins consisting of an anti-P-selectin single chain antibody (scFv) targeting domain linked to the complement inhibitor, Crry. Construction, expression, purification and in vitro characterization of these constructs was described previously [22]. The proteins were stored at −80C, and once thawed stored under sterile conditions at 4C for up to 2 weeks. Before use, binding activity of purified proteins was confirmed by standard ELISA using recombinant mouse P-selectin-Ig (BD Biosciences) as capture antigen and anti-His tag Ab (Clontech) for detection (data not shown). Complement inhibitory activity of constructs was confirmed by flow cytometric analysis of complement deposition in a standard zymosan assay [23].

## Treatment Paradigm

Two treatment paradigms were utilized: a sub-acute treatment paradigm (up to P14) and a chronic treatment paradigm (up to P45). For both paradigms, 2.12Psel-Crry, 2.3Psel-Crry or PBS was injected ip on P4 at 1 hour post injury at a dose of 20 mg/kg. For the acute timepoint treatment paradigm, treatments were administered at P4, 7, 10 and 13 for a total of 4 doses. For the chronic treatment paradigm, treatments were administered at P4, 7, 10, and 13 as above, and thereafter weekly up to P41 for a total of 7 doses.

## Germinal Matrix Hemorrhage Injury Model And Lesion Grading System

The GMH injury model and lesion grading system utilized was as we previously described [5]. Briefly, Clostridium-derived collagenase (Type VII-S collagenase, C2399–1.5 KU, Sigma-Aldrich) was injected into the SVZ of mouse pups at P4 to induce direct spontaneous non-traumatic vessel rupture with intracerebral hemorrhage in the region of the germinal matrix and SVZ. Sham PBS injections were performed to ensure that hemorrhage was a result of collagenase injection and not from mechanical insertion of the needle or dynamics of a fluid injection. The animal-specific injury grading system was used that established a distinction between parenchymal injury, ventricular involvement, and PHH. The grading system has been described [5], and is as follows: 0 = No lesion or ventricular enlargement; 1 = Lesion volume < 30% of hemispheric cortical tissue ipsilateral to injury site without ventricular involvement; 2 = Lesion volume > 30% of hemispheric cortical tissue ipsilateral to injury site without ventricular involvement; 3 = Lesion extending into the ipsilateral ventricle with no ventricular enlargement; 4 = Lesion extending into the ipsilateral ventricle coupled with unilateral ventriculomegaly; 5 = Lesion extending into both ventricles resulting in bilateral ventriculomegaly (global hydrocephalus).

## Behavioral Tests

### Nesting Building

Nest building task was performed at P40 as previously described [24]. Briefly, test mice were singly housed, and a new nestlet introduced to the cage 1 hr before the active phase (dark phase). The next morning, the remaining compacted cotton from the nestlet was weighed (untorn nestlet), and the nests scored on a rating scale of 1–5 in a blinded fashion. A well-structured nest is scored 5 and failure to disturb the nestlet is scored 1.

### Elevated Plus Maze

Mice were introduced in the center of the elevated plus maze (Stoelting #60140) in white light (100 lux) and recorded for 5 minutes using ANY-maze behavior tracking software (Stoelting) with center-point detection. Testing was performed at P42. Data are reported as the percent of time spent in the open areas.

### Fear-Conditioning

Fear conditioning was performed as previously described [25]. Training was performed at P44 and testing at P45. Briefly, test mice were placed in a fear conditioning chamber (MedAssociates) and allowed to explore the arena for 3 min, after which a loud auditory stimulus (30 s; 90dB) that co-terminates with a 2 s mild foot-shock (0.5 mA) is presented to the animal. This is repeated twice, with a 1 min interval separating the tones/shocks. After 24 hr, animals are returned to the chamber, and mouse behavior (moments of freezing and moving) when exposed to the same environment (contextual fear-conditioning) and when exposed to a new environment where the audible tone is played, is recorded by a video-tracking system (Video Freeze V2.6; MedAssociates). Data are presented as percent of time the mouse is immobile.

### Ultrasonic Vocalization (USV) Recordings

Distress USVs were recorded from juvenile mice as previously described [26]. Briefly, pups were recorded in a random order in a small, sound-attenuated chamber following separation from dam and littermates. USVs were recorded for 3 min on P5, 7, and 11 using Avisoft UltraSoundGate equipment (UltraSoundGate 116Hb with Condenser Microphone CM16; Avisoft Bioacoustics, Germany). USVs were analyzed using Avisoft SASLab Pro (Avisoft Bioacoustics) using a 20 kHz cutoff.

## Tissue Processing And Histological Analyses

Animals in the acute studies were sacrificed at P14. Following euthanasia, cardiac perfusion was performed with cold PBS followed by 4% paraformaldehyde mixed in PBS. Brains were extracted and placed in 4% paraformaldehyde solution overnight at 4°C. The brains were then moved to a new vial with 30% sucrose mixed with 4% paraformaldehyde in PBS. For tissue cutting, the brains were embedded in Tissue-Plus Optimal Cutting Temperature (OCT) compound (23–730-571, Fisher Healthcare) and frozen. Brains were cut in 40 μm coronal sections using a freeze-mount cryostat. Sections from the complete brain were collected in 12-well plates kept in PBS-filled wells until histologic analysis. For Nissl staining, serial brain sections 200 μm apart were mounted on a slide and stained using cresyl violet as previously described [27]. For ventricular and lesion volume measurements, 8 serial Nissl-stained brain sections 200 μm apart and 40 μm thick were used to reconstruct the total lesion volume. 4x magnification images of each slice were acquired using a Keyence BZ-X710 microscope (Keyence Co., Itasca, IL, USA). Lesion and ventricular areas were calculated using NIH ImageJ (FIJI) in a blinded fashion.

## Immunofluorescence Staining And Imaging

Mid-ventricular regions were identified by stereometric measurement using a mouse brain atlas, followed by standard (IF) staining as previously described [9]. All imaging and analysis were performed by lab personnel blinded to experimental samples. For microglia and complement IF staining, high-resolution imaging was performed using a Zeiss LSM 880 confocal microscope (Zeiss, Carl Zeiss Microscopy, LLC, White Plains, NY, USA) at 40x with water-media overlay and using the Z-stacking feature for Iba1 and C3 staining. Images were deconvoluted and reconstructed in 3D plane using Imaris Microscopy Image Analysis Software. Mean Fluorescent Intensity of 3D reconstructed image was quantified as total voxel number. For microglial morphology analyses and quantification of microglia-internalized complement (C3), high-resolution IF imaging was performed using a Zeiss LSM 880 confocal microscope at 63x with oil overlay and using the Z-stacking feature for Iba1 staining. A representative periventricular region was selected from each brain within naïve, vehicle, and treatment groups – with each region containing approximately 5 full intact microglia per Z-stack image. Individual microglia from 63x confocal images were then processed and analyzed using Imaris Microscopy Image Analysis Software as previously described [28]. For morphological analysis, microglial branch length, number of branches, and number of terminal points were identified on each cell manually and connected using the “FilamentTracer” tool (Imaris). The resultant tracings were used to calculate the total process length, number of processes, and number of terminal branch points. 3D rendering of microglia was achieved using Imaris “Surface” tool alongside the Imaris Labkit Analyses. Following reconstruction of microglia utilizing Channel Masking Technology (described in the Imaris Image Analysis Software instructions reference manual), C3 IF was reconstructed and then overlayed and masked with the reconstructed microglia to image internalized C3. C3 IF was then quantified per microglia as per Imaris Image Analysis Software instructions. For P-selectin analysis, 20x images of each section were acquired using a Keyence BZ-X710 microscope specifically at periventricular, hippocampal, and white matter brain regions. P-selectin was quantified by calculating the total integrated density (product of Area and the average signal intensity per pixel as a Mean Gray Value) using NIH ImageJ. All staining included negative control images (using secondary antibodies only) in order to correct for any underlying auto-fluorescence. Fluorescence-based analysis was performed rather than cell counting due to high cell density and clumping in the proximity of the injury site.

Primary antibodies used for staining were: anti-C3 (Abcam, Cat. #: ab11862, 1:200), anti-Iba1 (Invitrogen, Cat. #: PA5–21274, 1:200), and anti-CD62p (R&D, Cat. #: AF737, 1:200). Secondary antibodies utilized were all donkey and were anti-rabbit Alexa Fluor 488 nm (Invitrogen, Cat. #: A-21206, 1:200), anti-rat Alexa Fluor 488 nm (Invitrogen, Cat. #: A-21208, 1:200), anti-rat Alexa Fluor 555 nm (Abcam, Cat. #: ab150154, 1:200), anti-rabbit Alexa Fluor 555 nm (Invitrogen, Cat. #: A-31572, 1:200), anti-goat Alexa Fluor 647 nm (Invitrogen, Cat. #: A32849, 1:200).

## Tail Clipping Assay

Tail bleed time in P13 C57BL/6J pups was measured 2 hours after their designated final treatment (on P13) of PBS, 2.3Psel-Crry or 2.12Psel-Crry by a procedure previously described [22]. Briefly, pups were anesthetized with ketamine/xylazine mix and were placed in a prone position and a distal 5mm segment of the tail amputated. The tail was immediately immersed in pre-warmed isotonic saline at 37°C and each animal monitored till cessation of bleeding. If bleeding on/off cycles occurred, the sum of bleeding times within the 20-minute period was used.

## Flow Cytometry

Blood was collected from P14 mice by cardiac puncture at time of euthanasia in 50mM EDTA containing Futan-75 to prevent complement activation. Collected samples were centrifuged for 10 min at 600g. Supernatant was removed, and pellets resuspended in 1 mL citrate buffer containing 50 ng/mL PGE2 to prevent platelet activation. The resuspended samples were centrifuged for 10 min at 3,200g. Supernatant was then discarded and erythrocytes lysed using ACK buffer (ACK Lysing Buffer, Gibco, ThermoFisher Scientific) for 4 minutes. The samples were then washed with PBS for 10 min at 3,200g. Supernatant was removed and samples containing circulating immune cells and platelets were resuspended in FACS buffer, incubated with anti-FcR antibody (clone 24G2) and stained with the following primary antibodies: Ly6G (clone 1A8), Ly6C (clone AL-21), CD11b (clone M1/70, eBioscience), CD62P (clone RB40.34) and CD41 (clone MWReg30). All antibodies were purchased from BD Pharmingen. After staining samples were washed twice in FACS buffer, fixed for 10 minutes at 4°C in 1% paraformaldehyde, washed and resuspended in FACS buffer. The samples were acquired on a Fortessa X20 (BD Pharmingen) and analyzed with FlowJo software (TreeStar).

## Live Animal Fluorescence Tomography

2.3Psel-Crry was labeled with a fluorescent marker (CF dye 92221, Biotium, Fremont, CA, USA) per the manufacturer’s protocol and administered ip to P4 pups at 1 h after injection of collagenase or PBS. Live animal fluorescence tomography (Maestro II PerkinElmer, Waltham, MA, USA) was performed at 6 h, 24 h, 48 h, and 72 h after administration of fluorescently labeled 2.3Psel-Crry. Relative 2.3Psel-Crry brain deposition was quantified by measuring signal intensity within the brain using NIH ImageJ (FIJI) integrated density.

### Statistical analysis

Statistical analysis and data representation was achieved using GraphPad Prism 8.0 (GraphPad Software, San Diego, CA, USA). Details of statistical tests used for different analyses are described in figure legends. All data in the manuscript are represented as mean ± SEM and P values < 0.05 were considered significant. Power sample size estimation was done as previously stated based on prior work from our team and with an acceptable power range of 80–90% [5].

## Results

### Upregulation of P-selectin expression after induction of GMH

**Figure 1:**
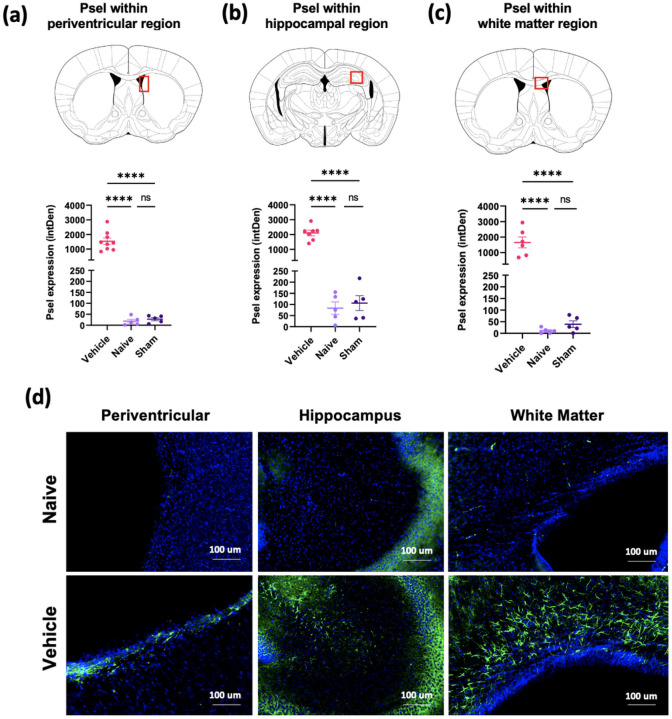
Expression of P-selectin in different brain regions following GMH. P14 brain sections stained for P-selectin (CD62p) and quantified in 3 specific brain regions of each experimental group as indicated (**a, b, c**). One-way ANOVA with Turkey’s correction for multiple comparisons. ****p< 0.0001. Error bars = mean ± SEM. (**d**) 40x representative images of P-selectin staining (green) and DAPI (blue) within each brain region for naïve and GMH animals.

Periventricular leukocyte infiltration is associated with GMH and its inflammatory pathological sequelae, although the expression of endothelial adhesion molecules has not been investigated. Using our neonatal murine GMH model involving intraventricular injection of collagenase at postnatal day 4 (P4), we demonstrated marked upregulation of P-selectin expression at 10 days after GMH (P14) (Fig. 1). P-selectin expression was evident post-GMH along the lesion border of ipsilateral ventricles, within the hippocampus, and in white matter tracts such as the corpus callosum. These data provide a rationale for exploring the use of P-selectin as a potential therapeutic target to treat GMH, and taken together with our previous data demonstrating a role for complement in the pathological sequelae of [5], we investigated an approach of P-selectin targeted complement inhibition. Specifically, we characterized the effect of 2.12Psel-Crry and 2.3Psel-Crry constructs in our GMH model, which are anti-P-selectin scFv targeting vehicles linked to the complement inhibitor Crry; the 2.12 scFv, but not the 2.3Psel scFv, additionally blocks P-selectin function (PSGL-1 binding).

## Effect of 2.12Psel-Crry and 2.3Psel-Crry on hydrocephalus development after GMH

**Figure 2:**
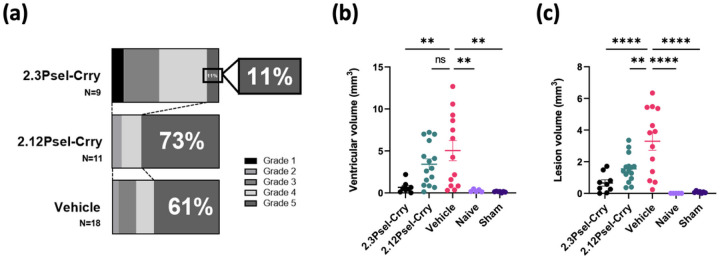
Treatment of GMH mice with 2.3Psel-Crry, but not 2.12Psel-Crry, reduces lesion size and hydrocephalus. P14 serial brain sections from indicated experimental groups were nissl stained and quantified for injury grade and ventricular and lesion volume. (**a**) Injury grade at P14 for 2.3Psel-Crry (n=9), vehicle (n=19) and 2.12Psel-Crry (n=11) treated GMH mice. (**b**) ventricular volume and (**c**) lesion volume quantification for each experimental group. One-way ANOVA with Sidak’s correction for multiple comparisons. **p<0.01, ****p<0.0001. Error bars = mean ± SEM.

Following induction of GMH in P4 pups, animals were treated with one of the fusion constructs in therapeutic paradigms (see methods), and lesion size and hydrocephalus outcomes evaluated at P14. Using a previously described GMH injury grading scale of 1–5 [5], 61% of vehicle treated animals developed Grade 5 global hydrocephalus compared with only 11% of animals treated with 2.3Psel-Crry (Fig. 2a). Unexpectedly, animals treated with 2.12Psel-Crry were not protected from hydrocephalus development and trended worse than vehicle treated controls, with 73% of 2.12Psel-Crry treated mice developing Grade 5 global hydrocephalus. Lesion and ventricular volumes were also quantified in each group from serial Nissl-stained sections. Both ventricle and lesion volume were reduced with 2.3Psel-Crry treatment compared to vehicle (Fig. 2b,c). By comparison, in 2.12Psel-Crry treated animals there was no difference in ventricular volume compared to vehicle, although there was a decrease in lesion volume. There was no significant difference between naïve and sham (PBS in place of collagenase injection) groups, indicating that induction of GMH was not simply the result of a mechanical injury. Thus, 2.3Psel-Crry significantly reduced ventricular volume and post-hemorrhagic hydrocephalus (PHH) after GMH induction, whereas 2.12Psel-Crry failed to provide protection.

## Effect of 2.12Psel-Crry and 2.3Psel-Crry on post-GMH inflammation

**Figure 3:**
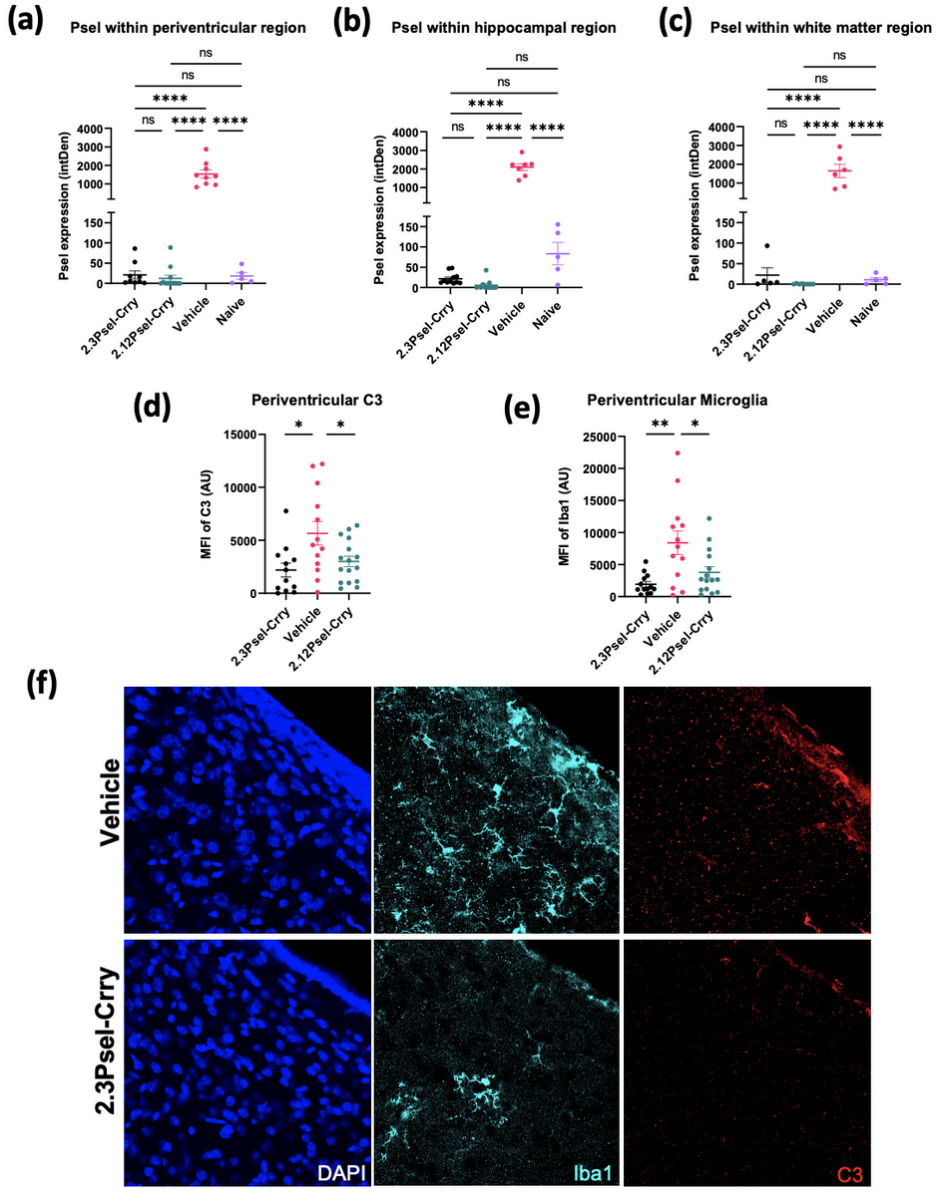
Effect of P-selectin targeted complement inhibition on P-selectin expression, microgliosis and complement deposition in GMH mice. P14 brains sections were stained by immunofluorescence microscopy for P-selectin in 3 specific brain regions and expression quantified: (**a**)ipsilateral periventricular region, (**b**) ipsilateral hippocampus, and (**c**)ipsilateral white matter (corpus callosum). Following immunofluorescence microscopy, C3 deposition (**d**) and microgliosis (**e**) was quantified in periventricular region of brains from indicated experimental groups. One-way ANOVA with Turkey’s correction for multiple comparisons. ****p< 0.0001. Error bars = mean ± SEM. (**f**) 63x representative images of Iba1 (teal) and C3 (red) within periventricular region of vehicle and 2.3Psel-Crry treated GMH mice.

Endothelial P-selectin is a marker and propagator of inflammation, and we first investigated the effect of the 2.12 and 2.3 constructs on P-selectin expression in the brain following GMH. P-selectin expression was analyzed within periventricular, hippocampal, and white matter tissue 10 days after GMH induction (P14). Consistent with data shown in Fig. 1, P-selectin expression was evident post-injury along ipsilateral ventricles and within the hippocampus and corpus callosum of vehicle treated GMH mice; however, little or no P-selectin expression was detected in any brain region from mice treated with either the 2.3 or the 2.12 construct (Fig. 3a–c). On a technical note, it is unlikely that the administered 2.3 or 2.12 constructs blocked binding of the anti-P-selectin detection antibody in this experiment, since the detection antibody was a polyclonal antibody, and also the 2.3 and 2.12 constructs do not bind to the same epitope.

In models of adult traumatic brain injury and ischemic stroke, complement activation leads to an aberrant process of microglial phagocytosis of complement opsonized neurons and synapses [9, 12]. There is also evidence that complement-dependent microglial phagocytosis may be involved in neuronal loss after GMH [5]. We therefore investigated the effect of 2.12Psel-Crry and 2.3Psel-Crry on C3 deposition and microglia recruitment. For analysis of microgliosis and its relationship to complement activation, brain sections from P14 mice were stained for Iba-1 and C3. (Fig. 3d–f). There was a significant decrease in C3 deposition within periventricular regions following treatment with both 2.3Psel-Crry or 2.12Psel-Crry compared to treatment with vehicle (Fig. 3d). Correlating with reduced C3 deposition, both Psel-Crry constructs resulted in decreased periventricular microgliosis compared to vehicle controls (Fig. 3e).

**Figure 4:**
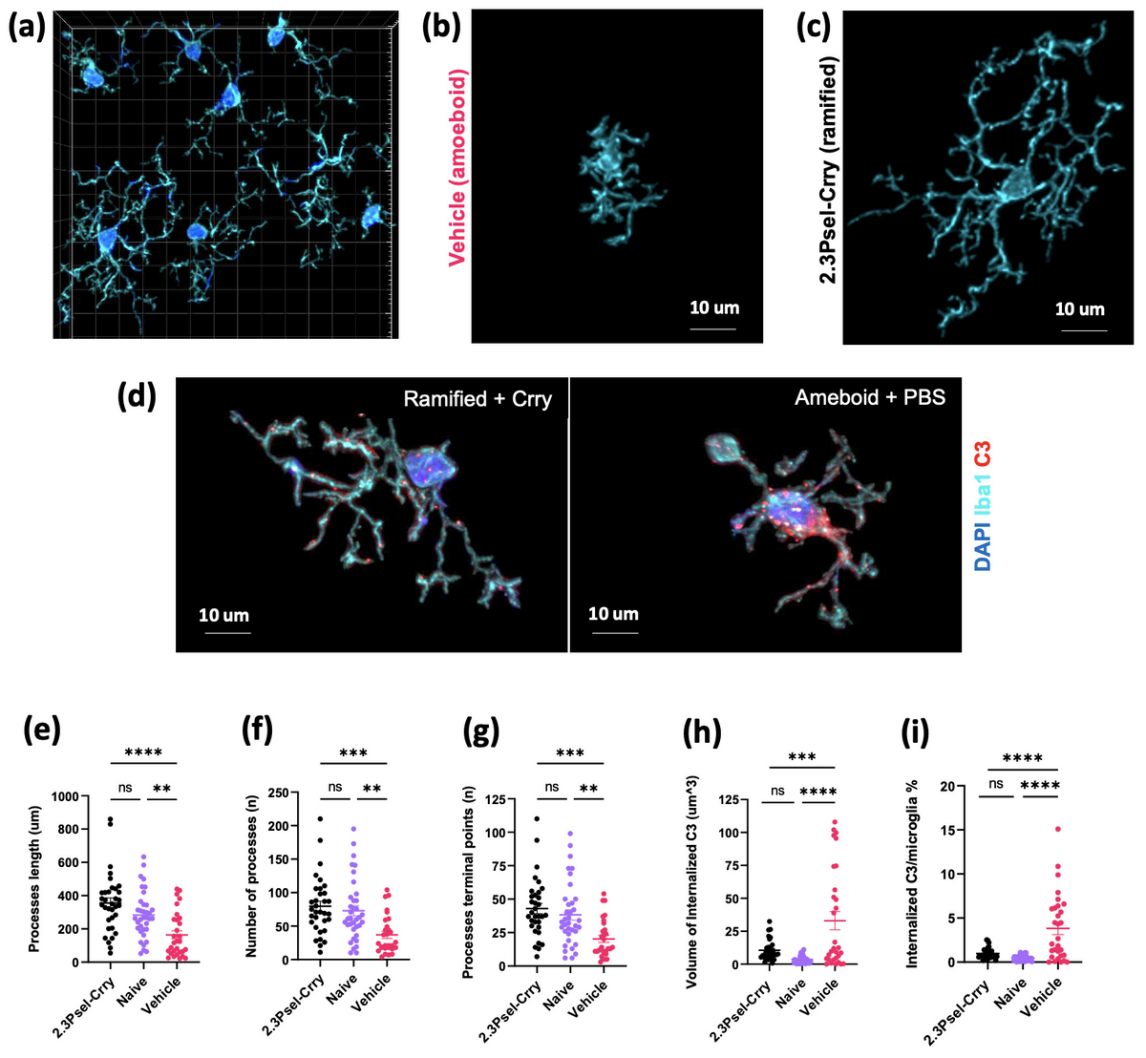
Microglial morphology and internalization of complement within the periventricular region after GMH. Morphological analysis of microglia from different experimental groups in terms of ramified vs. amoeboid characteristics. (**a**) Representative 63x confocal anti-iba1 immunofluorescent imaging following deconvolution and reconstruction for Imaris microglia processing. (**b**) Representative microglia from vehicle treated GMH mouse showing amoeboid characteristics. (**c**) Representative microglia from 2.3Psel-Crry treated GMH mouse treated mouse showing more ramified morphology.(**d**) Representative image of low level C3 internalization in a ramified microglia from 2.3Psel-Crry treated GMH mice (left) and high level C3 internalization in more ameboid-like microglia from vehicle treated GMH mice (right). Various morphologic characteristics were quantified: (**e**) summation of processes length per microglia, (**f**) summation of number of processes per microglia, and (**g**) summation of number of processes terminal points per microglia. One-way ANOVA with Turkey’s correction. *p< 0.05, **p< 0.01, ***p< 0.001, ****p<0.0001. Error bars = mean ± SEM. Internalization of complement deposition was quantified: (**h**) as total volume of internalized C3 and internalized (**h**) C3 volume within each microglia as a percentage of the individual microglia volume. One-way ANOVA with Sidak’s correction. ***p< 0.001, ****p< 0.0001. Error bars = mean ± SEM.

To further characterize the post-GMH microglial response, both with and without complement inhibition, we investigated microglial morphology within the periventricular region (Fig. 4). For these studies we focused only on the 2.3Psel-Crry construct given the lack of protection provided by 2.12Psel-Crry in this model. Microglial ramification can be used as a morphological measure to evaluate activation status and can be quantified in terms of the number and length of microglial processes. In general terms, highly ramified microglia are considered representative of resting/surveilling microglia, and a more ameboid form representative of a more activated status. Compared to microglia in the periventricular region of naïve animals, microglia in vehicle treated GMH animals displayed an overall decrease in process length as well as a decrease in the number of processes per individual microglia, thus displaying more ameboid characteristics (Fig. 4b,e,f) (Additional file 1). In comparison, microglia from 2.3Psel-Crry treated GMH mice displayed a more ramified morphology which was similar to microglial morphology in naïve animals (Fig. 4c,e,f) (Additional file 2). As an additional measure, vehicle treated GMH animals also displayed a decrease in number of terminal points as compared to 2.3Psel-Crry treated and naïve animals (Fig. 4g).

Finally, we investigated whether post-GMH pathology is associated with microglial uptake of C3 deposits, as has been shown with regard to C3 opsonization of neurons and synapses with other types of (adult) brain injury [9, 12]. Compared to microglia from naïve mice, in vehicle treated GMH mice there was increased microglial internalization of C3 in terms of increased total volume in analyzed sections, as well as increased C3 volume as a percent in individual microglia (Fig. 4d,h,i)(Additional file 3,4). This increase in C3 internalization by microglia was restored to levels seen in naïve mice when GMH animals were treated with 2.3Psel-Crry.

## Effect of 2.12Psel-Crry and 2.3Psel-Crry on coagulation

**Figure 5:**
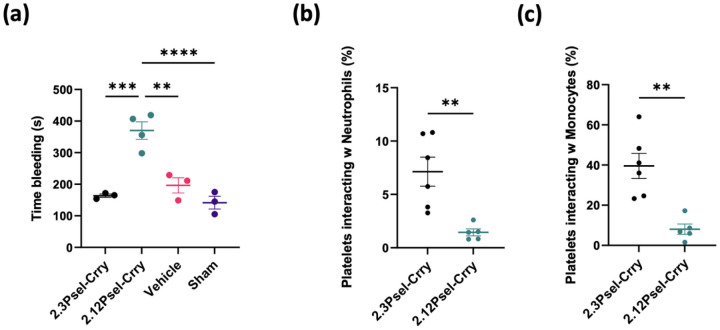
2.12Psel-Crry, but not 2.3Psel-Crry, has anti-coagulative properties. Compared to treatment of GMH mice with 2.3Psel-Crry or PBS, treatment with 2.12Psel-Crry increases bleeding time (**a**) and inhibits heterotypic platelet-leukocyte aggregation (**b,c**). Heterotypic platelet interactions with neutrophils and monocytes quantified by CD41+CD62P+CD11b+Ly6G+ populations and CD41+CD62P+CD11b+Ly6C+ populations via flow cytometry for each experimental group. Unpaired Student’s t-test. ** p < 0.01. Error bars = mean ± SEM. For (a), one-way ANOVA with Turkey’s correction. **p< 0.01, ***p< 0.001, ****p< 0.0001. Error bars = mean ± SEM.

We recently reported that following hindlimb ischemia and reperfusion in adult mice, 2.12Psel-Crry improved limb perfusion and increased bleeding time [22]. We thus considered that the difference in outcome between the 2.12 and 2.3 constructs in this hemorrhagic condition (2.12Psel-Crry worsens outcome and 2.3Psel-Crry improves outcome) may be related to an anti-coagulative effect of 2.12Psel-Crry. Indeed, we found that 2.12Psel-Crry, but not 2.3Psel-Crry, increased bleeding time in P14 pups compared to vehicle treated GMH mice or sham mice (Fig. 5a). To provide a potential mechanistic basis for this finding, we investigated the effect of each construct on heterotypic platelet-neutrophil (CD41 + CD62p + Ly6G+) and platelet-monocyte (CD41 + CD62p + Ly6C+) interactions in P14 pups. In this context, such heterotypic interactions are known to contribute to thrombus formation via the binding of platelet expressed P-selectin to PSGL-1 expressed on leukocytes [29, 30]. We found that 2.12Psel-Crry, but not 2.3Psel-Crry, significantly reduced both platelet-neutrophil and platelet-monocyte interactions (Fig. 5b,c), thus providing a likely explanation for the anti-coagulative effect of 2.12Psel-Crry and its effect on post-GMH outcomes.

## The effect of 2.3Psel-Crry on chronic outcomes after GMH

**Figure 6:**
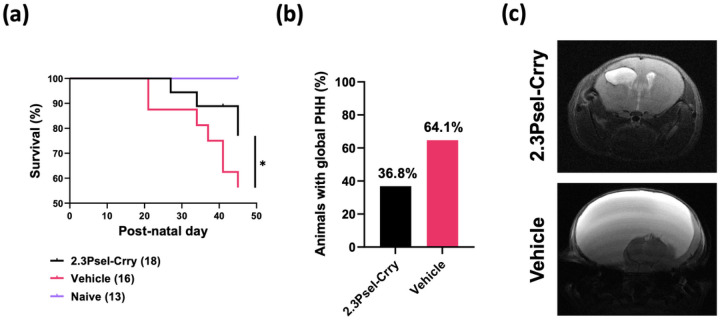
Long-term treatment of GMH mice with 2.3Psel-Crry improves survival and reduces white matter loss. **(a)** Survival assessed over 41 days after GMH injury (to P45) of different experimental groups, showing increase survival rates of GMH mice with 2.3Psel-Crry treatment. Log-rank (Mantel-Cox) test. *P<0.0205. Error bars = mean ± SEM. (**b**) Percent total number of animals with global PHH at P30 (when about 90% of animals in both groups are surviving) showing roughly only 35% of 2.3Psel-Crry treated animals developed PHH. (**c**)Representative MR imaging of 2.3Psel-Crry treated GMH mice with Grade 3 injury and vehicle treated GMH mice with Grade 5 injury.

We investigated the effect of 2.3Psel-Crry on chronic outcomes after GMH in terms of survival, hydrocephalus development, and cognitive function (2.12Psel-Crry was not investigated since it was not protective in this model). 2.3Psel-Crry treatment significantly improved survival of GMH mice compared to vehicle treatment (Fig. 6a). At experimental endpoint (P45), 2.3Psel-Crry treated mice had a nearly 80% survival rate, whereas only 55% of vehicle treated animals survived. Survival began to decline after P21, which is the time when animals are weaned. Correlating with survival rate was the incidence of global PHH as determined by MRI at P30, with 2.3Psel-Crry treatment reducing the number of animals that developed global hydrocephalus. (Fig. 6b,c).

**Figure 7:**
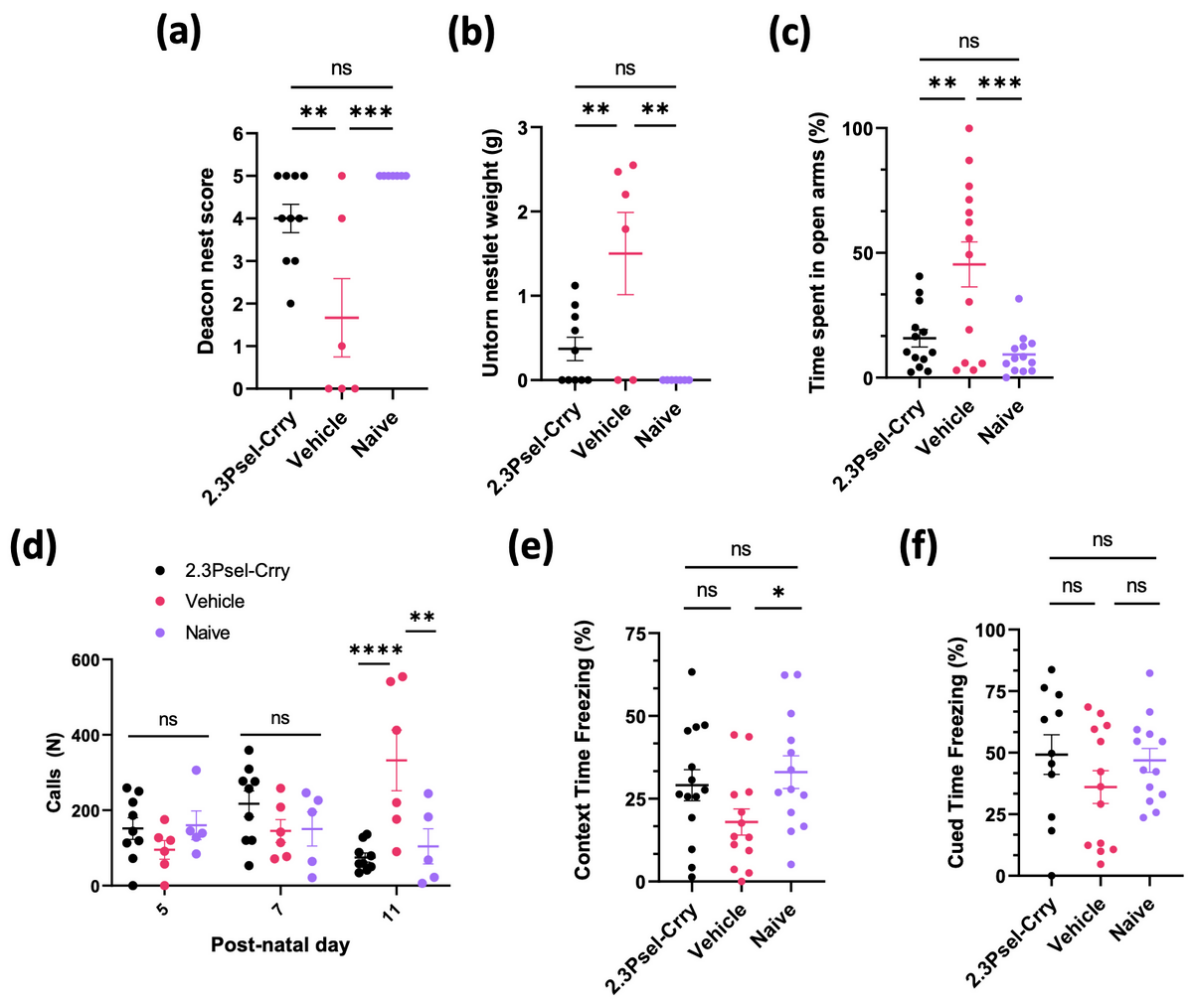
Behavioral analysis of GMH mice and effect of 2.3Psel-Crry treatment Nest Building task quantified by Deacon Nest Score (**a**) and weight of untorn nestlet square (**b**). (**c**)Elevated Plus Maze task quantified at P45 in terms of percent of time spent in open arms of maze. Increased stress and anxiety (more time in open arms) seen in vehicle treated GMH animals to 2.3Psel-Crry treated GMH animals. No difference between 2.3Psel-Crry treated and naïve mice. One-way ANOVA with Turkey’s correction. **p<0.01, ***p<0.001. Error bars = mean ± SEM. (**d**)Ultrasonic Vocalizations quantified by total number of calls when pups removed from mothers. Increased call number (increased stress and anxiety) in vehicle treated GMH mice compared to 2.3Psel-Crry treated and naïve mice at P11. Two-way ANOVA with Sidak’s correction for multiple comparisons. **p<0.01, ****p<0.0001. Error bars = mean ± SEM. (**e**) Contextual fear conditioning quantified at P45 by total percent of time freezing when re-exposed to an environment associated with a shock (no cue). Student’s t-test. *p<0.05. Error bars = mean ± SEM. (**f**) Cued fear conditioning quantified at P45 by total percent of time freezing after exposure to cue. Unpaired Student’s t-test. Error bars = mean ± SEM.

Taken together, the above data show that following GMH there is an ongoing complement-dependent neuroinflammatory response with the development of PHH and potential loss of white matter. We assessed whether this was linked to motor and cognitive deficits in adolescence (P45). The nest building task assesses changes in common social practices as well as cognitive and motor function. Compared to naïve mice, vehicle treated GMH mice performed significantly worse in this task, whereas 2.3Psel-Crry treatment restored nest building performance to that of naïve mice (Fig. 7a,b). Anxiety and stress were assessed using the elevated plus maze, where preference to being in open arms over closed arms is measure of anxiety-like behavior. Vehicle treated GMH mice showed increased exploration of the open arms of the maze compared to both 2.3Psel-Crry treated GMH mice and naïve animals, with no difference observed between 2.3Psel-Crry treated and naïve animals (Fig. 7c). Stress and anxiety were also measured at early time points in terms of ultrasonic vocalizations when pups were removed from mother and littermates. The number of ultrasonic calls emitted by a pup is used as an index of anxiety, and at P11, 2.3Psel-Crry treatment significantly reduced the number of vocalizations compared to vehicle treated animals and was indistinguishable from naïve animals (Fig. 7d). Hippocampal integrity and amygdala function was assessed via fear-conditioned memory retention with contextual and cued fear learning. When re-exposed to a shock environment (contextual stimulus), vehicle treated GMH mice spent significantly less time freezing compared to naïve mice. 2.3Psel-Crry treated mice showed a trend toward improvement compared to vehicle, although the difference did not reach significance. There was, however, no significant difference between 2.3Psel-Crry treated GMH mice and naïve mice (Fig. 7e), indicating that 2.3Psel-Crry may preserve neurocognitive ability and memory function. Finally, behavioral analyses of amygdala function following the presentation of a tone cue (conditioned stimulus) showed no significant differences between each group in terms of percent of time freezing during tone presentation (Fig. 7f). There has been little exploration into how GMH affects amygdala circuits, and it is difficult to draw further conclusion from this result.

## Targeting specificity of 2.3Psel-Crry after induction of GMH

**Figure 8:**
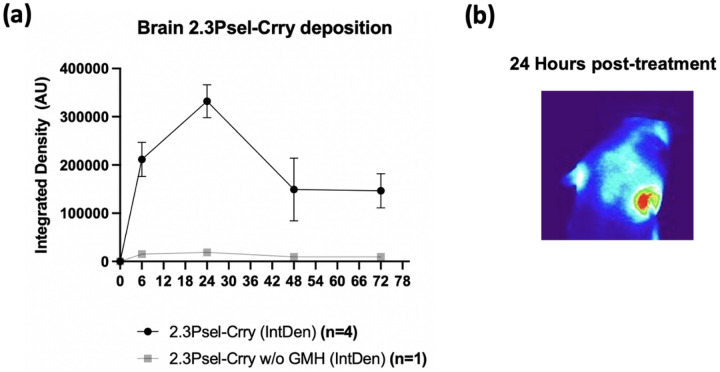
Localization of 2.3Psel-Crry to the post-GMH brain after intraperitoneal injection. 2.3Psel-Crry tagged with a fluorescent probe was administered i.p. to P4 pups 1 hour after induction of GMH or to a naïve (no injury) P4 animal (**a**) Quantification of fluorescence intensity by live animal fluorescence tomography of head at indicated time points after 2.3Psel-Crry administration. (**b**) Representative 24 hour fluorescence intensity image following administration of fluorescent-tagged 2.3Psel-Crry. n=4 for 2.3Psel-Crry with GMH injury. n=1 for 2.3Psel-Crry without GMH injury.

To examine the targeting specificity of 2.3Psel-Crry after its systemic administration, we injected fluorescently labeled 2.3Psel-Crry via ip injection after induction of GMH. Live animal fluorescence tomography showed localization of 2.3Psel-Crry to the injury site (collagenase injection site) within the brain at 6 hours post injection, reaching peak intensity around 24 hours post injection (Fig. 8). Notably, a substantial fluorescence signal remained in the brain at 72 hours post injection. Together with our previous data showing a fast-phase circulatory half-life of less than 1 hour for 2.3Psel-Crry [22], the data highlight an important feature of this site-targeted inhibitor, namely long-lived localized complement inhibition at the site of injury, with a minimal and rapidly declining effect on systemic complement activity.

## Discussion

GMH is a hemorrhagic event in neonates that is characterized as having a primary insult that is untreatable. Furthermore, the greater severity of hemorrhage the greater the risk for morbidity and mortality, with a 90% rate of morbidity and mortality in severe cases [31]. While the primary insult is unpredictable and untreatable, the process of initiation and progression of secondary injury, such as the onset of PHH, represents a therapeutic target. Although the mechanisms leading to secondary injury and the development of PHH are not well understood, it is known that there is an increased inflammatory response post-GMH leading to hypersecretion of CSF by the choroid plexus, the recruitment of inflammatory cells, and increased recruitment of astrocytes [32–34]. The complement system plays a central role in inflammatory responses, and complement has recently been implicated in post-GMH sequelae and the propagation of PHH. In the setting of GMH and neonatal hypoxic-ischemic brain injury, complement activation has been associated with increased recruitment of microglia and astrocytes, neuronal engulfment, and development of PHH [5, 13, 14]. Thus, given the lack of treatment options for GMH and recent evidence indicating a role for complement in the propagation of post-GMH injury, it is of interest to explore complement inhibitory strategies as investigative tools as well as for their therapeutic potential.

The current study shows that following GMH, P-selectin expression is upregulated in periventricular, hippocampal, and white matter regions, which can be expected to contribute to the secondary inflammatory response following GMH, since P-selectin is a vascular adhesion molecule that participates in the recruitment of leukocytes. Of note, both 2.3Psel-Crry and 2.12Psel-Crry mitigated P-selectin upregulation, the former of which does not block P-selectin adhesion function. In this regard, we have previously shown that complement inhibition alone (not targeted to P-selectin) can also mitigate P-selectin upregulation following brain injury [20]. Indeed, there is a dynamic relationship between P-selectin and complement in that complement activation products can upregulate P-selectin expression, and P-selectin can directly activate complement [17–21]. We also demonstrate herein that although only the nonblocking 2.3Psel-Crry is protective in terms of post-GMH lesion size and the development of PHH, both constructs reduced C3 deposition and microgliosis within perilesional areas.

Microglia perform canonical functions involved in normal fetal and neonatal brain development, as well as perform homeostatic functions such as the clearance of pathogens and cellular debris. However, microglia can also contribute to a damaging neuroinflammatory response when they become aberrantly activated [35, 36]. Complement can play a key role in microgliosis, and in particular aberrant phagocytosis of viable neurons and synapses during a secondary response after brain injury [12, 37, 38]. Activated microglia can also perpetuate an inflammatory response by activating neurotoxic reactive astrocytes [39]. Herein we show that C3 deposition is localized to areas of microgliosis after GMH, and that treatment of GMH mice with either of the targeted complement inhibitors ablates both C3 deposition and microgliosis within periventricular brain regions. We also linked complement activation to the transitioning of microglia to a more ameboid morphology, and demonstrated that microglia in 2.3Psel-Crry treated mice retain a more ramified morphology that is associated with a normal resting/surveilling phenotype. Furthermore, improved outcomes in GMH mice treated with 2.3Psel-Crry was associated with a significant reduction in uptake of C3 deposits by microglia.

In addition to the anti-inflammatory properties of both targeted complement inhibitors, the 2.12Psel-Crry construct exhibited anti-coagulative properties and interfered with heterotypic platelet/leukocyte aggregation. This hemodynamic effect of 2.12Psel-Crry almost certainly accounts for its lack of protection in our hemorrhagic model, in which the anti-inflammatory effect of the construct was unable to overcome the initial hemorrhagic insult made worse by 2.12Psel-Crry. It is clear that P-selectin has both pro-inflammatory and thrombogenic activities, having roles in immune cell infiltration, platelet aggregation and coagulation [29, 30], all of which are relevant to GMH and injury progression. And while a 2.12Psel-Crry type construct is obviously not a therapeutic candidate for GMH or other hemorrhagic conditions, it is noteworthy that complement activation is closely associated with multiple thrombotic conditions; for example various rheumatic and autoimmune conditions, transplant related conditions and renal microangiopathies [40–42]. Also in this context, we have shown that complement inhibition potentiates thrombolytic therapy with tissue plasminogen activator in a model of ischemic stroke [43].

Common pathological sequelae of GMH include the development of PHH and PVL, both of which can lead to major cognitive impairment. This has been shown in multiple clinical studies where there is a direct link between premature neonates having clinical grades 3–4 intraventricular hemorrhage with cerebral palsy (CP) and significant mental deficits. Indeed, even neonates with clinical grades 1–2 are at risk for developmental disability [44–47]. In our murine GMH model, we show that secondary injury post-GMH involves a progressive neuroinflammatory response that leads to the development of PHH and PVL. We also show that complement plays an important role in the initiation and progression of the inflammatory response that leads to PHH and PVL. Correlating with clinical findings, we show that the development of PHH in our model is associated with motor and cognitive impairment in adolescence, and that both of these outcomes are mitigated by treatment with 2.3Psel-Crry. Nest building is a behavioral task dependent on limbic function and models daily living activities that may be altered in patients with cognitive impairment [24, 48]. Nest building activity was significantly impaired in GMH mice compared to naive controls, and elevated plus maze data revealed higher levels of stress and anxiety in adolescent GMH mice. Stress and anxiety was also increased earlier in life in GMH mice as measured by resolution of hyper-vocalization when pups were removed from their mother. All of these behavioral outcomes were significantly improved in GMH mice treated with 2.3Psel-Crry, and there were no differences between GMH mice treated with 2.3Psel-Crry and naive animals. Hippocampal integrity and amygdala function was assessed via fear-conditioned memory retention with contextual and cued fear learning. Behavioral testing via contextual fear learning shows hippocampal functionality was decreased in vehicle treated GMH mice, and while there was a trend toward improvement with 2.3Psel-Crry treatment, the difference did not reach significance. Overall, these data indicate a complement-dependent progression of secondary injury after GMH leading to profound chronic alterations that result in cognitive dysfunction, and which is reversible by P-selectin targeted complement inhibition.

## Conclusions

Germinal matrix hemorrhage is the most common neurologic pathology in neonates and there is no current treatment for its pathological sequelae. Here we show that vascular P-selectin expression and complement activation within the brain are hallmarks of GMH modeled pathology. We show that complement is a key mediator of a neuroinflammatory response and the progression of secondary injury post-GMH, and that a P-selectin targeted complement inhibitor reduces post-GMH microgliosis and hydrocephalus occurrence, and preserves neurocognitive function later in life. Of the two P-selectin targeted complement inhibitors we characterized, one had an additional anti-coagulative property, which while detrimental for treating hemorrhagic conditions such as GMH, has therapeutic potential for treating neuroinflammatory pathologies that include a thrombotic event, such as ischemic stroke or indeed other non-CNS thrombotic pathologies in which complement plays a role. Finally, on a translational note, the 2.3Psel and 2.12Psel scFv targeting vehicles recognize both mouse and human P-selectin, and humanization of antibodies/antibody fragments is a standard technique. The human ortholog of Crry is CR1, and this protein has been shown to be safe in humans when used as an untargeted construct [49].

## Data Availability

All data generated and/or analyzed during the current study are included in this published article and the supplementary material. Materials described in this article that were made in the laboratory will be made available upon request under a materials transfer agreement.

## Abbreviations

GMH: Germinal matrix hemorrhage
SVZ: Subventricular zone
PHH: Post-hemorrhagic hydrocephalus
IVH: Intraventricular hemorrhage
TBI: Traumatic brain injury
P: Post-natal day
MAC: Membrane attack complex
CSF: Cerebrospinal fluid
IP: Intraperitoneal
